# Midlife blood pressure change and left ventricular mass and remodelling in older age in the 1946 British birth cohort study^†^

**DOI:** 10.1093/eurheartj/ehu389

**Published:** 2014-09-22

**Authors:** Arjun K. Ghosh, Rebecca J. Hardy, Darrel P. Francis, Nishi Chaturvedi, Denis Pellerin, John Deanfield, Diana Kuh, Jamil Mayet, Alun D. Hughes

**Affiliations:** 1 International Centre for Circulatory Health, National Heart and Lung Institute, Imperial College London, 59–61 North Wharf Road, London W2 1LA, UK; 2 Medical Research Council Unit for Lifelong Health and Ageing, University College London, London, UK; 3 The Heart Hospital, University College London, London, UK; 4 Institute of Cardiovascular Science, University College London, London, UK

**Keywords:** Blood pressure, Left ventricular mass, Left ventricular hypertrophy, Echocardiography

## Abstract

**Aims:**

Antecedent blood pressure (BP) may contribute to cardiovascular disease (CVD) independent of current BP. Blood pressure is associated with left ventricular mass index (LVMI) which independently predicts CVD. We investigated the relationship between midlife BP from age 36 to 64 and LVMI at 60–64 years.

**Methods and results:**

A total of 1653 participants in the British 1946 Birth Cohort underwent BP measurement and echocardiography aged 60–64. Blood pressure had previously been measured at 36, 43, and 53 years. We investigated associations between BP at each age and rate of change in systolic blood pressure (SBP) between 36–43, 43–53, and 53–60/64 years on LVMI at 60–64 years. Blood pressure from 36 years was positively associated with LVMI. Association with SBP at 53 years was independent of SBP at 60–64 years and other potential confounders (fully adjusted *β* at 53 years = 0.19 g/m^2^; 95% CI: 0.11, 0.27; *P* < 0.001). Faster rates of increase in SBP from 43 to 53 years and 53 to 60/64 years were associated with increased LVMI. Similar relationships were seen for diastolic, pulse, and mean pressure. Rate of increase in SBP between 43–53 years was associated with largest change in LVMI (*β* at 43–53 years = 3.12 g/m^2^; 95% CI: 1.53, 4.72; *P* < 0.001). People on antihypertensive medication (43 years onwards) had greater LVMI even after adjustment for current BP (*β* at 43 years = 12.36 g/m^2^; 95% CI: 3.19, 21.53; *P* = 0.008).

**Conclusion:**

Higher BP in midlife and rapid rise of SBP in 5th decade is associated with higher LVMI in later life, independent of current BP. People with treated hypertension have higher LVMI than untreated individuals, even accounting for their higher BP. Our findings emphasize importance of midlife BP as risk factor for future CVD.

**See page 3242 for the editorial comment on this article (doi:10.1093/eurheartj/ehu371)**

## Introduction

High blood pressure (BP) is a key risk factor for cardiovascular disease (CVD)^1^ and is associated with increased left ventricular (LV) mass (LVM)^2^ and left ventricular hypertrophy (LVH) and remodelling.^3^ LVH is widely used as a measure of target organ damage in hypertension and increased LVM and LVH are associated with CVD independent of current BP.^2^

Exposure to elevated BP over the life course increases the risk of CVD.^4^ There is evidence that earlier development of hypertension,^5^ elevated BP in early adulthood^6^ or antecedent elevated BP^7^ confer increased risk. This might reflect the relative stability (tracking) of BP across life; the cumulative burden of lifetime exposure to high BP; or vulnerability to the effects of high BP at particular periods in the life course, i.e. sensitive periods. The extent to which the influence of long-term elevated BP can be reversed by treatment or the extent to which long-term treatment prevents increases in LVM has been little studied outside the context of clinical trials, although several trials have demonstrated that antihypertensive treatment (HTT) causes regression of LVM in the short term.^8^

We studied the nationally representative 1946 British birth cohort [the Medical Research Council National Survey of Health and Development (MRC NSHD)], to determine the relationship between longitudinal changes in BP over a 28-year period (aged 36 to 60–64) and LVM at age 60–64 years and also assessed the impact of HTT in the same individuals over the same period.

## Methods

### Participants

The MRC NSHD is a prospective birth cohort study of singleton births that occurred in 1 week of March 1946 in England, Scotland, and Wales (5362 births; 2547 women, 2815 men). The parents of the cohort members were married with equal numbers of fathers engaged in manual and non-manual work. Follow-up included over 20 contacts with the whole cohort between birth and the most recent data collection when the participants were between 60 and 64 years of age. The study complies with the Declaration of Helsinki, the research protocol was approved by the local ethics committee and all participants gave informed consent.

Survey members had been interviewed in their own homes by trained research nurses at ages 36, 43, and 53 years.^9^ Between October 2006 and February 2011 (at 60–64 years), 2856 eligible study members (those known to be alive and with a known address in England, Scotland, or Wales) were invited to complete a postal questionnaire and attend an assessment at one of six Clinical Research Facilities (CRF). Invitations were not sent to those who had died (*n* = 778), were living abroad (*n* = 570), had previously withdrawn from the study (*n* = 594), or had been lost to follow-up (*n* = 564). Those who declined the clinic visit were offered a home examination by a trained nurse. Of those invited, 2229 (78%) responded: 1690 (59.1%) attended a CRF and 539 (18.9%) had a home visit.

### Clinic assessment

Height and weight were measured at the CRF and body mass index (BMI) calculated. Sitting brachial BP was measured in the upper right arm as the average of two measures with an appropriately sized cuff after 5 min of rest. At 36 and 43 years a Hawksley Random Zero sphygmomanometer was used and an Omron HEM-705 was used at 53 and 60–64 years. Measurements from the Random Zero sphygmomanometer were adjusted using published conversion equations to achieve compatibility with later measurements.^10^ Mean arterial pressure (MAP) was calculated as diastolic BP (DBP) + 0.33*pulse pressure (PP). Fasting bloods were also drawn for analysis.

### Echocardiographic studies

Of the 1690 participants who attended a clinic, 1653 (798 men and 855 women) underwent echocardiography performed by a trained, experienced sonographer according to a strict protocol using GE Vivid I machines and 1480 had analysable images (89%). We carried out sensitivity analyses excluding those with a history of prior myocardial infarction. As there were no significant change on exclusion (Supplementary material online, *Table S10*), only those with unanalysable images were excluded from the analyses. Echocardiographic images were obtained from parasternal long axis and short axis, apical 5-, 4-, 3-, 2-chamber and aortic views along with conventional and tissue Doppler in the 4-chamber view. Image optimization was carried out if required using second harmonic imaging. Image analysis was carried out by AKG along with two experienced echocardiographers masked to patient identity using GE EchoPac software (GE Connecticut, USA).

Wall and chamber measures were made in a single core laboratory, LVM was indexed (LVMI) to body surface area (BSA),^11^ alternative indexation (height^1.7^ and height^2.7^)^12,13^ was also performed to check robustness of findings in the setting of overweight or obesity. Calculation of variables and definition of LVH and LV remodelling was performed according to ASE/EAE guidelines.^11^ Quality assurance of echocardiography was performed throughout the study and blind duplicate reading reproducibility studies were carried out on a sample of studies (equal numbers of men and women and with varying image quality) to establish inter- and intrareader variability with excellent reproducibility (intraclass correlation coefficients > 0·8 for all measurements).

### Medication, antihypertensive treatment, and diabetes status

Medication use was recorded and classified according to the International Classification of Diseases and related Health Problems classification.^9,14^ Type 2 diabetes mellitus (T2DM) was diagnosed based on self-report or clinic blood tests.

### Statistical analysis

Regression models were used to investigate the association between BP [systolic blood pressure (SBP), DBP, PP, and MAP] at each of the 4 ages at which it was measured and LVMI at 60–64 years, routinely with pre-specified covariable adjustment for sex, age at CRF visit and CRF attended. All analyses were carried out with SBP, DBP, PP, or MAP as the respective measure of BP in the model. Interactions between each BP measure and sex were tested, but no significant interactions were found for any of the outcomes presented and data for men and women were pooled. Further multivariable modelling was performed with antihypertensive treatment (HTT), current SBP, BMI, T2DM, smoking status, and physical activity status as covariables. Regression diagnostics were performed, including checks of linearity by examination of residuals and by testing for model improvement on addition of a quadratic term. To investigate whether rate of change at a particular period of midlife was more strongly associated with LVMI, we calculated the change in SBP for the periods 36–43 years, 43–53 years, and 53–60/64 years conditional on earlier SBP by modelling each SBP measure (from 43 years onwards) on the earlier measure(s) for each sex and saving the residuals. These residuals reflect mean rate of change in SBP and can be interpreted as the change in SBP above or below that expected on average, given earlier SBP.^15^ The residuals were standardized to allow a comparison of the relative strength of associations between periods. We subsequently fitted regression models including all these standardized changes with LVMI or relative wall thickness (RWT, a measure of remodelling) as the outcome. The coefficients for each period were compared against each other using Wald tests and models were further adjusted for potential confounders. Relationships with LVH were analysed using multivariable logistic regression. Sensitivity analyses were carried out to assess whether the associations with BP remained unchanged if those on HTT or those who were hypertensive (SBP> = 140 mmHg and/or DBP > = 90 mmHg) were excluded.

**Figure 1:**
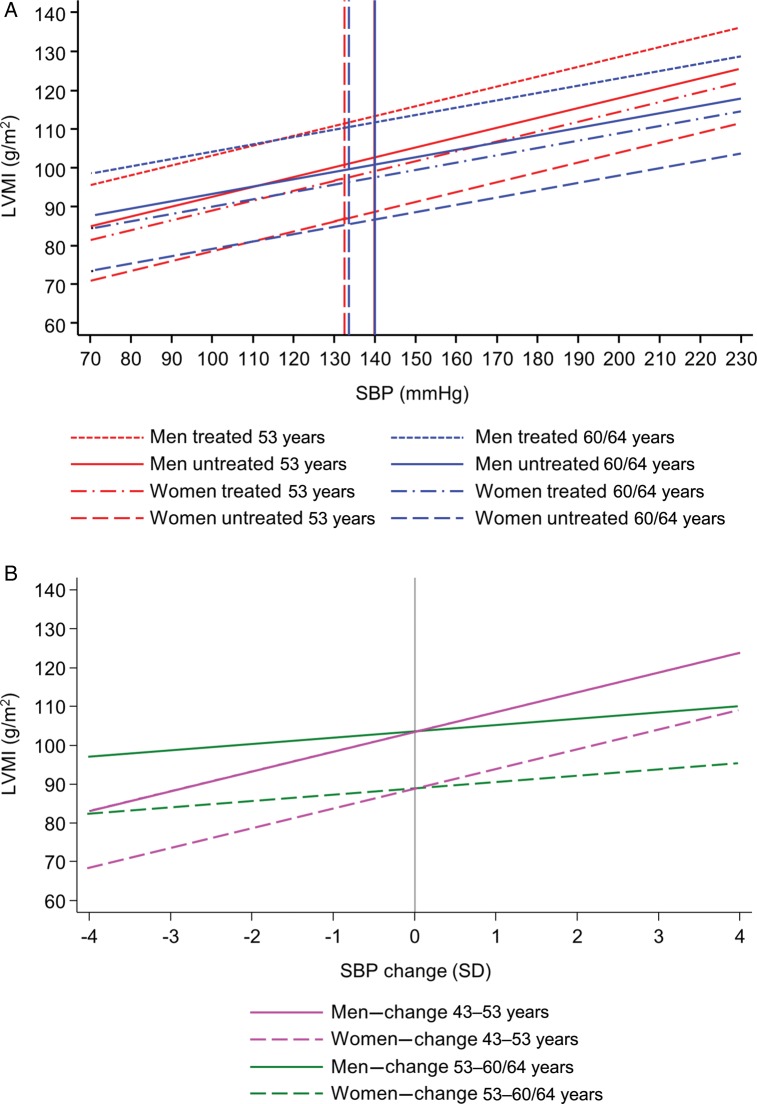
(*A*) Predicted left ventricular mass index at 60–64 years by systolic blood pressure at 53 years (red) and by systolic blood pressure at 60–64 years (blue) plotted separately for men and women and by antihypertensive treatment status. Vertical lines represent mean systolic blood pressure in the National Survey of Health and Development sample; solid line for men and dashed line for women. (*B*) Predicted left ventricular mass index at 60–64 years by standard deviation change in systolic blood pressure between 43 and 53 years (magenta) and between 53 and 60–64 years (green) plotted separately for men (solid) and women (dashed), for individuals with an average conditional change in the other periods. Vertical lines represent mean systolic blood pressure change.

## Results

Participant characteristics are shown in *Table 1*. Twenty-four percent of the sample was receiving antihypertensive medication (details of the class of antihypertensive agent used are shown in Supplementary material online, *Table S1*); 5% of the sample had T2DM; and a normal LV geometry was seen in 43%. Compared with those who attended for echocardiography, those who had home visits had higher BP (138.4/79.3 vs. 135.6/77.3 mmHg), BMI (28.7 vs. 27.6), and a greater prevalence of T2DM (11 vs. 5%) and hypertension (32 vs. 24%).

**Table 1:** Characteristics of study participants having at least one echocardiographic measure of interest recorded (*n* = 1480)

Variable	*N* men/women	Value (combined)	Value [men (*n* = 701)]	Value [women (*n* = 779)]
Age at echo (years)	701/779	63.3 (1.1)	63.2 (1.1)	63.3 (1.1)
Body mass index (kg/m^2^)	701/779	27.5 (4.6)	27.7 (4.0)	27.4 (5.1)
Left ventricular internal diameter in diastole (cm)	701/779	4.82 (0.6)	5.05 (0.6)	4.61 (0.5)
Interventricular septal thickness in diastole (cm)	701/779	1.1 (0.2)	1.1 (0.2)	1.0 (0.2)
Left ventricular posterior wall thickness in diastole (cm)	701/779	1.0 (0.2)	1.0 (0.2)	0.9 (0.2)
Left ventricular mass (g)	701/779	181.3 (59.3)	209.1 (60.4)	156.04 (45.7)
Left ventricular mass indexed to BSA (g/m^2^)	701/779	95.7 (26.6)	104.0 (28.0)	88.1 (23.0)
Left ventricular mass indexed to height^1.7^ (g/m^1.7^)	701/779	74.3 (22.5)	80.6 (23.5)	68.3 (20.0)
Left ventricular mass indexed to height^2.7^ (g/m^2.7^)	701/779	44.2 (13.2)	46.1 (13.7)	42.4 (12.6)
Relative wall thickness	701/777	0.42 (0.09)	0.42 (0.09)	0.41 (0.08)
Ejection fraction (%)	700/776	68.7 (9.7)	67.2 (10.1)	69.9 (9.2)
Left ventricular remodelling subtype (normal/concentric remodelling/ concentric hypertrophy/eccentric hypertrophy), *n* (%)	701/779	632 (43)/396 (27)/ 240 (16)/212 (14)	287 (41)/202 (29)/ 113 (16)/99 (14)	345 (44)/194 (25)/ 127 (16)/113 (15)
Systolic blood pressure at 60–64 years (mmHg)	701/777	135.7 (17.9)	139.0 (17.7)	132.8 (17.7)
Systolic blood pressure at 53 years (mmHg)	659/739	134.1 (19.1)	137.8 (18.9)	130.7 (18.7)
Systolic blood pressure at 43 years (mmHg)	655/730	121.5 (14.5)	123.0 (14.3)	120.1 (14.6)
Systolic blood pressure at 36 years (mmHg)	633/706	118.3 (14.0)	121.3 (14.2)	115.6 (13.4)
Diastolic blood pressure at 60–64 years (mmHg)	701/777	77.4 (9.7)	79.0 (9.8)	75.9 (9.3)
Diastolic blood pressure at 53 years (mmHg)	659/739	83.4 (11.9)	86.2 (11.9)	80.9 (11.3)
Diastolic blood pressure at 43 years (mmHg)	655/730	78.5 (11.3)	80.9 (11.2)	76.3 (10.9)
Diastolic blood pressure at 36 years (mmHg)	632/706	76.0 (11.7)	77.8 (11.7)	74.4 (11.4)
Heart rate (bpm)	701/777	68.5 (11.8)	67.2 (12.5)	69.7 (11.0)
Total cholesterol (mmol/L)	657/729	5.7 (1.2)	5.4 (1.1)	6.0 (1.2)
High-density lipoprotein cholesterol (mmol/L)	657/729	1.6 (0.4)	1.4 (0.3)	1.8 (0.4)
Fasting triglycerides (mmol/L)^a^	657/729	1.1 (0.8)	1.2 (0.9)	1.0 (0.7)
Fasting glucose (mmol/L)	667/740	5.8 (1.2)	6.0 (1.2)	5.6 (1.2)
Haemoglobin A1C (%)	656/731	5.8 (0.7)	5.8 (0.7)	5.8 (0.6)
Antihypertensive medication, *n* (%) on antihypertensive medication with controlled BP (<140/90 mm Hg), *n* (%)	620/712	315 (24)	159 (26)	156 (22)
		169 (53)^b^	79 (50)	90 (58)^b^
T2DM, *n* (%)	635/722	73 (5)	37 (6)	36 (5)

Data are mean (SD).

^a^Median (interquartile range) for skewed variables.

^b^One individual receiving antihypertensive medication was missing a valid measurement of blood pressure.

### Associations between left ventricular measures and blood pressure at various ages

Associations between BP and LV outcomes were qualitatively similar for all measures of BP (SBP, DBP, PP, and MAP), so results for SBP are shown below and associations for DBP, PP, and MAP are provided in Supplementary material online, *Table S2–S4*.

Higher SBP from 36 years onwards was associated with higher LVMI at 60–64 years (*Table 2*). Associations remained essentially unchanged after adjustment for HTT (Model 2). Systolic blood pressure at 53 years remained independently associated with LVMI with only marginal attenuation in an additional model that was adjusted for SBP at 60–64 years (Model 3), but associations with SBP at 36 years and particularly with SBP at 43 years were weakened. The relationships between LVMI and BP at 53 years and LVMI and HTT at 53 years remained after further adjustment for BMI, presence of T2DM, smoking and physical activity status at 60–64 years. A 10 mmHg higher SBP at 53 years was associated with a 2.2 g/m^2^ higher LVMI after adjustment for age, sex, CRF attended, HTT, and SBP at 60–64 years.

**Table 2:** Regression between left ventricular mass index at 60–64 years and systolic blood pressure and antihypertensive treatment at four time points with further adjustment for covariables

Independent variable		Model 1		Model 2		Model 3		Model 4	
		*β* (95% CI)	*P*	*β* (95% CI)	*P*	*β* (95% CI)	*P*	*β* (95% CI)	*P*
Age 36 (*n* = 1165)	SBP	0.18 (0.06, 0.29)	0.003	0.18 (0.06, 0.29)	0.003	0.12 (0.00, 0.24)	0.051	0.13 (0.01, 0.24)	0.032
Age 43 (*n* = 1199)	SBP	0.16 (0.05, 0.27)	0.003	0.15 (0.04, 0.26)	0.006	0.08 (−0.04, 0.19)	0.187	0.08 (−0.03, 0.19)	0.150
Age 53 (*n* = 1220)	SBP	0.30 (0.22, 0.37)	<0.001	0.25 (0.18, 0.33)	<0.001	0.22 (0.14, 0.30)	<0.001	0.19 (0.11, 0.27)	<0.001
Age 60–64 (*n* = 1276)	SBP	0.20 (0.13, 0.28)	<0.001	0.19 (0.11, 0.27)	<0.001	NR	–	0.16 (0.08, 0.24)	<0.001
Age 36	HTT	1.66 (−12.89, 16.21)	0.823	0.35 (−14.18, 14.87)	0.963	1.33 (−13.10, 15.75)	0.857	−0.82 (−14.84, 13.20)	0.908
Age 43	HTT	16.72 (7.31, 26.13)	<0.001	16.00 (6.60, 25.39)	0.001	16.19 (6.86, 25.53)	0.001	12.36 (3.19, 21.53)	0.008
Age 53	HTT	13.80 (9.55, 17.90)	<0.001	10.49 (6.21, 14.77)	<0.001	10.65 (6.37, 14.93)	<0.001	8.63 (4.36, 12.91)	<0.001
Age 60–64	HTT	11.59 (8.35, 14.82)	<0.001	11.15 (7.94, 14.36)	<0.001	NR	–	8.84 (5.56, 12.13)	<0.001

The variable *β* is regression coefficients for LVMI vs. SBP (mmHg) or HTT.

CI, confidence interval. Model 1: adjusted for age sex and CRF attended. Model 2: Model 1 + HTT at given age (for SBP) or Model 1 + SBP at given age (for HTT). Model 3: Model 2 + SBP at 60–64 years. Model 4: Model 3 + T2DM + BMI + smoking status + physical activity status. NR, not relevant.

These associations remained when those with hypertension or on HTT were excluded from analyses (Supplementary material online, *Table S5 and S6*, respectively), although associations with earlier measures of SBP were slightly reduced.

Relationships between BP (both SBP and DBP) and RWT showed a similar pattern to those between BP and LVMI. There was evidence of associations of BP from 43 years onwards with RWT which were independent of BP at 60–64 years (*Table 3*; Supplementary material online, *Table S7*).

**Table 3:** Regression between relative wall thickness at 60–64 years and systolic blood pressure and antihypertensive treatment at four time points with further adjustment for covariables

Independent variable		Model 1		Model 2		Model 3		Model 4	
		*β* (95% CI) × 10^3^	*P*	*β* (95% CI) × 10^3^	*P*	*β* (95% CI) × 10^3^	*P*	*β* (95% CI) × 10^3^	*P*
Age 36 (*n* = 1163)	SBP	0.15 (−0.25, 0.54)	0.469	0.15 (−0.24, 0.54)	0.455	0.03 (−0.37, 0.43)	0.886	0.04 (−0.37, 0.44)	0.863
Age 43 (*n* = 1197)	SBP	0.68 (0.32, 1.05)	<0.001	0.67 (0.30, 1.04)	<0.001	0.57 (0.18, 0.95)	0.004	0.58 (0.20, 0.97)	0.003
Age 53 (*n* = 1219)	SBP	0.54 (0.28 0.79)	<0.001	0.51 (0.24, 0.77)	<0.001	0.42 (0.14, 0.71)	0.004	0.38 (0.10, 0.67)	0.009
Age 60–64 (*n* = 1274)	SBP	0.41 (0.13, 0.68)	0.004	0.38 (0.11, 0.66)	0.006	NR	–	0.32 (0.04, 0.60)	0.024
Age 36	HTT	−9.05 (−58.14, 40.03)	0.718	−10.17 (−59.35, 39.01)	0.685	−8.13 (−57.21, 40.95)	0.745	−17.31 (−66.23, 31.60)	0.488
Age 43	HTT	22.30 (−10.61, 55.21)	0.184	18.95 (−13.85, 51.74)	0.257	19.47 (−13.30, 52.25)	0.244	12.17 (−20.64,44.97)	0.467
Age 53	HTT	14.55 (−0.35, 29.45)	0.056	7.97 (−7.24, 23.17)	0.304	8.38 (−6.83, 23.59)	0.280	4.01 (−11.44, 19.46)	0.611
Age 60–64	HTT	14.51 (3.21, 25.81)	0.012	13.65 (2.36, 24.94)	0.018	NR	–	6.73 (−4.99, 18.44)	0.260

The variable *β* is regression coefficients for RWT vs. SBP (mmHg) or HTT. CI, confidence interval. Model 1: adjusted for age sex and CRF attended. Model 2: Model 1 + HTT at given age (for SBP) or Model 1 + SBP at given age (for HTT). Model 3: Model 2 + SBP at 60–64 years. Model 4: Model 3 + T2DM + BMI + smoking status + physical activity status. NR, not relevant.

LVH was also associated with BP from 36 years onwards, although only weakly with BP at 43 years. The association at 53 years was independent of BP at 60–64 years and was unaffected by adjustment for other covariables (for SBP: odds ratio = 1.25, 95% CI = 1.07, 1.45 per 1 SD increase in SBP at 53 years adjusted for age, sex, CRF attended, HTT, SBP at 60–64 years, T2DM, BMI, smoking, and physical activity).

### Associations between left ventricular measures and antihypertensive treatment at various ages

Individuals on HTT from 43 years onwards had a higher mean LVMI than those who were not on treatment (*Table 2*); this difference persisted after adjusting for SBP at 60–64 years and other covariables. For example, individuals receiving HTT at 60–64 years had an LVMI that was 11.2 g/m^2^ greater than those not receiving treatment. Further adjustment for SBP at 60–64 years and other covariables reduced the associations slightly.

We investigated the possibility that the relationship between LVMI and HTT could be explained by people receiving treatment at 60–64 years including a disproportionate number of individuals with high BP at 53 years. However, adjustment for SBP at 53 years only slightly attenuated the effect of HTT on LVMI from 11.2 (95% CI: 7.9, 14.4) g/m^2^–7.9 (95% CI: 4.3, 11.4) g/m^2^. Adjustment for SBP at younger ages had negligible effects.

### Evidence for a rate-sensitive period in midlife

Faster increases in SBP between 43–53 and 53–60/64 years were significantly related to greater LVMI (*Figure 1*), whereas increased rate of change in SBP between 36 and 43 years was only weakly related to LVMI. The relationship between rate of change in SBP between 43 and 53 years was significantly stronger than the other two intervals (*P* = 0.002 for both). After adjustment for HTT, T2DM, BMI, smoking status, and physical activity (*Table 4*), the associations with change in the latest two periods remained, although the association between change in SBP between 43 and 53 years was somewhat weakened. Estimates were essentially unaltered when further adjusted for prior measures of BMI or changes in BMI (Supplementary material online, *Table S8*).

**Table 4:** Relationship between LVMI at age 60–64 years and rate of change in systolic blood pressure at three time periods (36–43 years), (43–53 years), and (53–60 to 64 years) on LVMI (*n* = 1085)

Period of rate of change in systolic blood pressure	Model 1		Model 2	
	*β* (95% CI)	*P*	*β* (95% CI)	*P*
36–43 years	1.38 (−0.25, 3.01)	0.096	0.42 (−1.20, 2.04)	0.612
43–53 years	5.10 (3.53, 6.67)	<0.001	3.12 (1.53, 4.72)	<0.001
53–60/64 years	1.62 (0.10, 3.15)	0.037	1.87 (0.35, 3.38)	0.016

The variable *β* is regression coefficients for LVMI (g/m^2^) for a 1 SD increase in systolic blood pressure in each interval. CI, confidence interval. Model 1: adjusted for age, sex, and CRF attended. Model 2: Model 1 + T2DM + BMI + smoking status + physical activity status + current HTT.

### Analyses with left ventricular mass indexed to height^1.7^ or height^2.7^

All analyses were repeated with LVM indexed to height^1.7^ and height^2.7^. Associations were qualitatively similar to those for LVM indexed to BSA. The associations between SBP and DBP at various ages and alternatively indexed LVM are presented in Supplementary material online, *Table S9 and S10*.

## Discussion

Elevated BP from early midlife predicts higher LVMI, LVH, and LV remodelling in older men and women independent of current BP. We identified 43–53 years as a potential sensitive period when rapid rises in SBP may be particularly detrimental for the development of adverse cardiac structure. We also found that people receiving HTT had higher LVMI, even after accounting for current BP; only some of this effect could be accounted for by prior higher BP.

A few previous studies have investigated the relationship between antecedent high BP in earlier life on subsequent LVM. Urbina *et al.*^16^ reported a significant positive correlation between SBP at baseline in young women but not young men (average age 13 years) and LVM measured ∼4 years later. Toprak *et al.*^17^ failed to find a significant relationship between SBP measured at 13 years and LV geometry in adulthood, although high DBP in childhood was associated with subsequent concentric LVH. Dekkers *et al.*^18^ found that PP and SBP measured in early adolescence correlated with LVM measured in African American and European American youths 10 years later; and Ridderstrale *et al.*^19^ reported that high BP in a selection of hypertensive male military recruits and controls aged ∼20 was predictive of higher LVM 20 years later. Two other studies have reported that BP in middle age was positively associated with LVM measured two decades later.^20,21^ The majority of these studies did not examine whether the effect of antecedent BP was independent of current BP and therefore the possibility that these observations were simply a consequence of a high correlation between antecedent and current BP (i.e. tracking) could not be excluded. However, a longitudinal analysis of the Framingham offspring study^22^ reported that BP in adulthood (mean age 45 years) correlated with the trajectory of LVM over a subsequent 16 years period.

Our findings based on multiple longitudinal measurements of BP extend these earlier studies and show that the effects of antecedent BP in adulthood are not wholly attributable to tracking of BP. Notably, BP at 53 years was more strongly related to LVMI and RWT than BP at 60–64 years and remained significant in a mutually adjusted model suggesting that midlife BP is more influential for LV structure than BP at age 60–64. After adjustment for current BP a 1 SD increase in BP at age 53 years was associated with a 4.2 g/m^2^ higher LVMI, and a 1 SD higher BP at age 53 years was associated with a 25% increased odds of LVH at age 60–64 years. These associations persisted even after multivariable adjustment for other risk factors including BP at age 60–64 years. We further show that a more rapid BP rise between 43–53 years is the strongest correlate of elevated LVMI, with a 1 SD higher rate of rise in BP in this period being associated with a 5.1 g/m^2^ greater LVMI. The increases in LVM associated with antecedent BP and rate of rise of BP are likely to have prognostic implications: an ∼5 g/m^2^ higher LVMI (∼0.2 SD of LVMI) can be predicted to correspond to a 7–20% increase in CVD morbidity and mortality on the basis of previous outcome studies.^23,24^ The importance of BP at 53 years as a predictor of LVMI may reflect the relatively rapid acceleration in BP which typically precedes this age.^25^ We analysed rate of change in BP over a 28-year period and divided it into temporal segments to establish if there was a ‘sensitive’ period when exposure to elevated BP had a particularly strong influence.^15^ Our data suggest that the 5th decade of life (∼40–50 years) may be such a period. This is consistent with previous data relating lifetime risk of CVD to BP and BP change in middle age.^5^ It is also interesting to note that both elevated BP and an exaggerated rise in midlife have also previously been reported to be risk factors for dementia two to three decades later, regardless of subsequent BP.^26,27^

Our finding that people with treated hypertension had higher LVMI, even after adjustment for current BP, is in keeping with a previous study showing that LVMI and prevalence of LVH was greater in individuals with well-controlled BP than in normotensive individuals.^28^ This led the authors to suggest that effective treatment of hypertension may not achieve complete reversal of cardiac target organ damage. The question of the part played by regression of LVM in risk reversal by HTT is debated.^29^ However, we speculate that elevated BP in midlife causes a ‘legacy’ of cardiac target organ damage including interstitial and perivascular fibrosis that may be difficult to reverse even if BP is subsequently well controlled with medication.^30^ This provides an additional rationale for on-going therapeutic efforts targeted at reversal of myocardial fibrosis.^31^ While elevated LVM predicts coronary events and cardiac failure,^2^ whether the failure to achieve normalization of LVM implies a subsequent excess of CVD and heart failure remains to be established. Planned future follow-ups of the British MRC NSHD cohort will provide important information on this question.

Current hypertension guidelines do not take into account antecedent BP or changes in BP when initiating therapy.^32,33^ Our data suggest that this may miss an opportunity to prevent cardiac target organ damage and its sequelae (e.g. heart failure) in later life. Recent randomized clinical trials have examined the short-term effect of antihypertensive agents in preventing development of hypertension in high-risk individuals with conflicting results.^34,35^ However, we are unaware of any studies exploring the possible longer-term benefits of BP lowering in individuals with pre-hypertension or an elevated rate of rise in BP in midlife. We suggest this may be a fruitful area of future clinical research.

### Strength and limitations

Participants in the MRC NSHD have previously been found to be representative of native-born adults living in England, Scotland, and Wales at the time of data collection.^9^ Hence, our findings are likely to be generalizable to the native-born British population. The repeated measurements of BP in our study, the longest running birth cohort in Britain, allowed us a rare opportunity to carry out a longitudinal analysis investigating importance of BP and rate of change in BP over different periods of adulthood. Missing data are inevitable in studies as long running as the MRC NSHD;^14^ however, response and attendance rates were acceptable. Compared with study members who had echocardiography, those who had examinations at home had higher BP and were less healthy, consistent with previous work.^36^ Exclusion of this less healthy group from our analyses may have resulted in an underestimation of the strength of associations between BP and LV structure. Blood pressure was not measured in the NSHD prior to 36 years, and hence we were unable to assess whether there are important periods in earlier life when BP may influence cardiac structure. We are also limited to identifying three potential sensitive periods of ∼10 years duration; hence, we may have underestimated the extent of BP change and/or the importance of more rapid rises in BP over shorter-time periods than the ∼10 years sampling intervals. The lack of 24 h ambulatory measurements of BP in this cohort also precludes us from examining whether short-term variability or diurnal variation in BP contributes to the associations seen. While we examined a range of potential confounders, unmeasured confounders, or mediators that influence cardiac hypertrophy (e.g. renin–angiotensin system activity, sympathetic nervous system activity, abnormalities of lipid metabolism, inflammation, etc.) may have contributed to the associations observed.^29^ Echocardiography was only carried out in the most recent round of data collection and hence the possibility of reverse causality, namely that increased LVM in early-life results in raised BP and increased rate of rise in midlife BP cannot be excluded. However, a previous study by Zureik *et al.*^37^ demonstrated that change in BP preceded change in LVMI, arguing against this explanation.

## Conclusions

Higher BP in midlife and, in particular, a rapid rate of BP increase in the 5th decade is associated with higher LVM and LV remodelling in later life; this association is independent of current BP. People with treated hypertension have higher LVMI than untreated individuals, even when accounting for their higher BP. Our findings emphasize the importance of midlife BP as risk factor for future CVD.

## Supplementary material

Supplementary material is available at *European Heart Journal* online.

## Funding

This work is supported by Medical Research Council UK, National Institute of Health Sciences Biomedical Research Centre Award to Imperial NHS Healthcare Trust, and The National Institute for Health Research University College London Hospitals Biomedical Research Centre. Funding to pay the Open Access publication charges for this article was provided by the Medical Research Council.

## 

**Conflict of interest**: None declared.
